# Getting the right cells

**DOI:** 10.7554/eLife.80373

**Published:** 2022-06-30

**Authors:** Bilal Cakir, In-Hyun Park

**Affiliations:** 1 Department of Genetics, Yale Stem Cell Center, Child Study Center, Yale School of Medicine New Haven United States

**Keywords:** brain organoid, brain vasculature, neural progenitors, blood-brain-barrier, microglia, None

## Abstract

Fusing brain organoids with blood vessel organoids leads to the incorporation of non-neural endothelial cells and microglia into the brain organoids.

**Related research article** Sun XY, Ju XC, Li Y, Zeng PM, Wu J, Zhou YY, Shen LB, Dong J, Chen Y, Luo ZG. 2022. Generation of vascularized brain organoids to study neurovascular interactions. *eLife* **11**:e76707. doi: 10.7554/eLife.76707.

Brain organoids, also known as brain spheroids, are three-dimensional cultures of neural cells derived from pluripotent stem cells (PSCs) that mimic the brain’s organization, development, and activity in a dish (Lancaster and Knoblich, 2014). The technology available to develop brain organoids has advanced considerably in recent years, providing an unprecedented opportunity to examine brain development and disease (Pasca, 2018). However, existing brain organoids still have limitations: most notably, they do not contain vascular cells, immune cells and other non-neural cells.

The neurovascular system is responsible for the delivery of oxygen and nutrients to the brain, the growth and development of neural tissue, and allowing the brain to perform its roles normally (Jin et al., 2002). Thus, a functional vasculature is critical to obtaining brain organoids with an architecture similar to the mature brain, and cells that have differentiated appropriately.

Several strategies have been devised to generate vascularized brain organoids (Figure 1). The simplest and most straightforward method is to co-culture brain organoids with endothelial cells which line blood vessels (Pham et al., 2018). Another approach is to genetically induce blood vessels by expressing a transcription factor that converts PSCs into endothelial cells as the brain organoids develop (Cakir et al., 2019). Yet another way to obtain vascularized brain organoids is to fuse PSC-derived brain organoids with endothelial cell organoids (Song et al., 2019).

**Figure 1.**
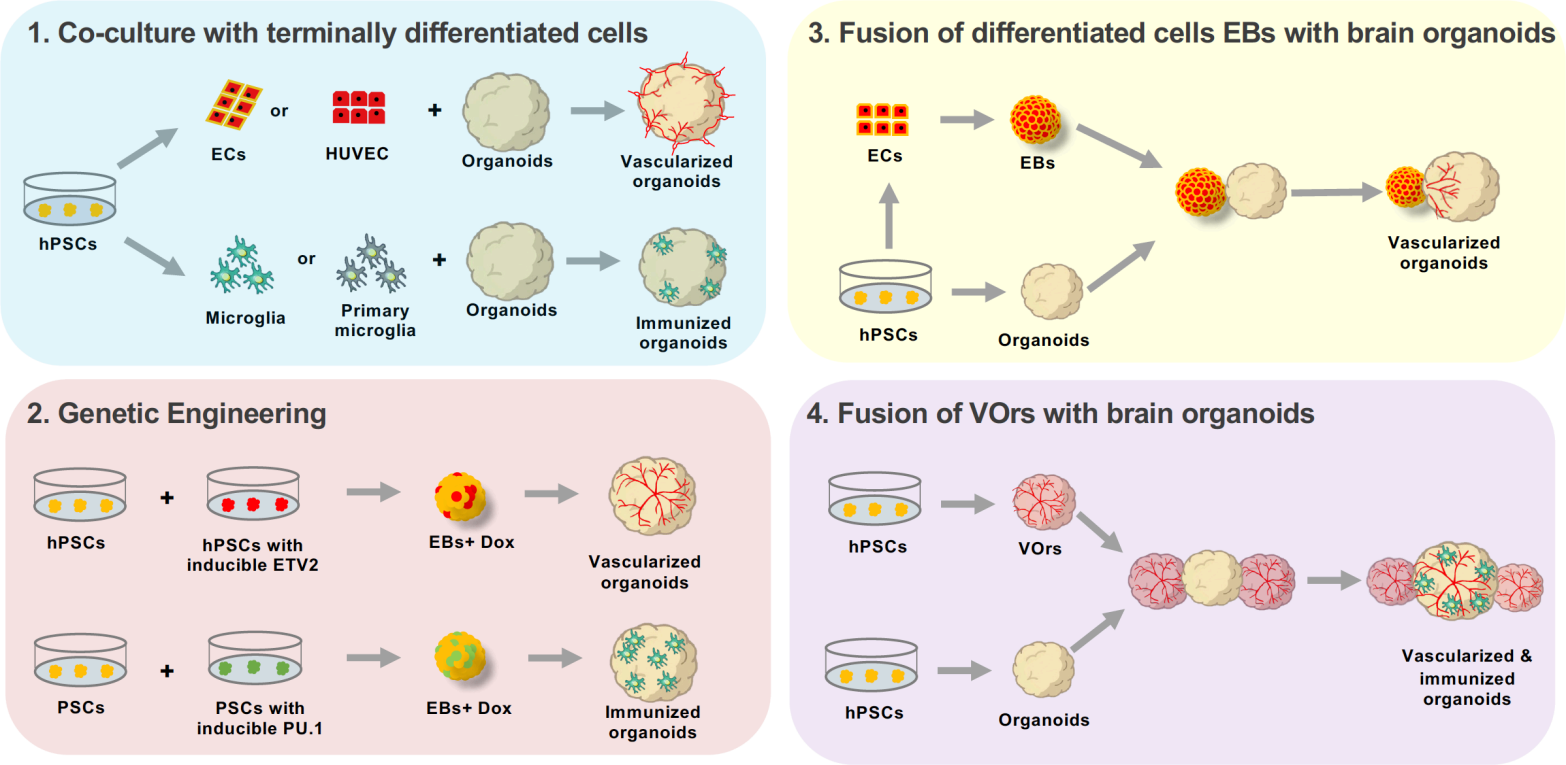
Approaches to enhance cellular diversity of brain organoids. (1) A co-culture approach consists on growing brain organoids alongside endothelial cells (ECs, red) or microglia (green). The endothelial cells and microglia can be obtained through differentiation from human pluripotent stem cells (hPSCs, yellow, differentiating into ECs or microglia; left), or by isolating the cells from the tissue of interest (center). Endothelial cells can be isolated from the umbilical vein (human umbilical vein endothelial cells or HUVEC, top center), while microglia are obtained by isolating the cells directly from the brain (primary microglia, bottom center). Co-culturing these differentiated cells with organoids leads to either vascularized organoids (when using endothelial cells, top right) or immunized organoids (when using microglia, bottom right). (2) Genetic engineering can be used to induce the working vessels and immune cells within brain organoids. To do this, unmodified human pluripotent stem cells (hPSCs, yellow, left) are mixed with hPSCs that have been genetically engineered to over-express a specific transcription factor when doxycycline is applied (center left). Cells carrying ETV2 (red, top) will differentiate into endothelial cells when doxycycline is applied, while cells carrying PU.1 (green, bottom) will differentiate into microglia. The wild-type cells are mixed with the genetically modified cells to form embryoid bodies (EBs) to which doxycycline is applied (center right). The embryoid bodies with cells that overexpress ETV2 develop into vascularized organoids (top right), while the embryoid bodies with cells that overexpress PU.1 develop into immunized organoids (bottom right). (3) Brain organoids can also be vascularized by fusing them with endothelial cell spheroids. In this case, hPSCs (bottom left) are differentiated into either endothelial cells (top left) or aggregated into organoids (bottom center). The endothelial cells are then aggregated into an endothelial spheroid (top center), which is then cultured alongside the organoid to generate a vascularized organoid (right). (4) Sun et al. have developed a new protocol for generating brain organoids with a vascular-like system. This protocol uses hPSCs to make vessel organoids (top) and brain organoids (bottom). Each brain organoid is then co-cultured with two vessel organoids. The organoids then fuse, leading to vascularized brain organoids that have microglia-like cells.

Similar strategies have been applied to add microglia, another non-neural cell type, to brain organoids (Abud et al., 2017; Xu et al., 2021; Cakir et al., 2022). Although each method has its limitations, all aim to incorporate a single type of non-neural cells into brain organoids. Now, in eLife, Zhen-Ge Luo from ShanghaiTech University, Xiang-Chun Ju from the Chinese Academy of Sciences, and co-workers – including Xin-Yao Sun as first author – report on the generation of fusion vessel brain organoids (fVBOrs) with both vascular- and microglia-like cells obtained by fusing vessel organoids with brain organoids (Sun et al., 2022).

Sun et al. first focused on generating vessel organoids. To do this, they activated Wnt signaling in embryonic bodies made of human embryonic stem cells (hESCs). The Wnt signaling pathway triggers hESCs to differentiate into mesodermal cells, which give rise to muscles, blood vessels, and connective tissue during development. Once the cells in the embryoid bodies had differentiated into the mesoderm, Sun et al. added vascular endothelial growth factor (VEGF) to further differentiate them into endothelial cells.

At this point, the embryoid bodies were embedded in Matrigel, a culture substrate containing many extracellular matrix components that cells encounter in vivo. Next, Sun et al. added neurotrophic factors – molecules that promote the growth and maturation of neurons – to the embryoid bodies to reproduce the brain trophic environment and facilitate the differentiation of the vessel organoids into brain vessels. As a result, the vessel organoids acquired complex vascular structures.

The next step was to characterize these vessel organoids by performing single-cell RNA sequencing (scRNA-seq). The results showed that the vessel organoids have characteristics unique to the vascular system, including the expression of groups of genes that are match the ones active in endothelial cells, vascular progenitors, fibroblasts, pericytes, and smooth muscle cells. Interestingly, a type of immune cells in the brain called microglia was also detected in these vessel organoids, likely due to the neurotrophic factors in the maturation media.

Once the vessel organoids had been developed and characterized, Sun et al. applied a co-culture approach to develop brain organoids with vascular systems (Figure 1). First, they made brain organoids using unguided protocols of intrinsic neuroectoderm differentiation, where hESCs are cultured together in signal-free media and spontaneously differentiate into neural cells (Lancaster and Knoblich, 2014). In previous studies, a single brain organoid had been fused with a single vessel organoid to achieve vascularization (Song et al., 2019). In the current study, however, each brain organoid was fused with two vessel organoids within Matrigel to generate ffVBOrs by entirely surrounding the neural tissue with the invading vascular structures.

Notably, neural tissue from these fVBOrs also contained functional microglia-like cells, which take part in the maturation of neural networks by engulfing synapses and regulating neuronal activity. Therefore, incorporating vascular and immune cells into the brain organoids might further promote the survival of neuronal progenitors by providing them with the growth factors they need. However, the spontaneous and stochastic differentiation of mesodermal progenitors into different cell types in vessel organoids can lead to high variability in the generation of microglia in fVBOrs, which could become an issue.

Despite the potential limitations, the fVBOrs reported by Sun et al. offer an excellent opportunity to examine the interaction between neural tissue and vascular structures during early brain development. Notably, this model differs from previously published approaches by acquiring endothelial and immune cells, the major non-neural cells missing in brain organoids. In the future, this study could be extended into other organoid models that mimic specific regions of the human brain and require a greater cellular diversity.
